# Mean computed tomography value to predict spread through air spaces in clinical N0 lung adenocarcinoma

**DOI:** 10.1186/s13019-024-02612-2

**Published:** 2024-04-23

**Authors:** Marino Yamamoto, Masaya Tamura, Ryohei Miyazaki, Hironobu Okada, Noriko Wada, Makoto Toi, Ichiro Murakami

**Affiliations:** 1 Department of Thoracic Surgery, Kochi Medical School, Kohasu, Oko-Cho, Nankoku, Kochi 783-8505 Japan; 2 Department of Pathology, Kochi Medical School, Nankoku, Kochi Japan

**Keywords:** Lung adenocarcinoma, Spread through air spaces, Mean-computed tomography value

## Abstract

**Background:**

The aim of this study was to assess the ability of radiologic factors such as mean computed tomography (mCT) value, consolidation/tumor ratio (C/T ratio), solid tumor size, and the maximum standardized uptake (SUVmax) value by F-18 fluorodeoxyglucose positron emission tomography to predict the presence of spread through air spaces (STAS) of lung adenocarcinoma.

**Methods:**

A retrospective study was conducted on 118 patients those diagnosed with clinically without lymph node metastasis and having a pathological diagnosis of adenocarcinoma after undergoing surgery. Receiver operating characteristics (ROC) analysis was used to assess the ability to use mCT value, C/T ratio, tumor size, and SUVmax value to predict STAS. Univariate and multiple logistic regression analyses were performed to determine the independent variables for the prediction of STAS.

**Results:**

Forty-one lesions (34.7%) were positive for STAS and 77 lesions were negative for STAS. The STAS positive group was strongly associated with a high mCT value, high C/T ratio, large solid tumor size, large tumor size and high SUVmax value. The mCT values were − 324.9 ± 19.3 HU for STAS negative group and − 173.0 ± 26.3 HU for STAS positive group (*p* < 0.0001). The ROC area under the curve of the mCT value was the highest (0.738), followed by SUVmax value (0.720), C/T ratio (0.665), solid tumor size (0.649). Multiple logistic regression analyses using the preoperatively determined variables revealed that mCT value (*p* = 0.015) was independent predictive factors of predicting STAS. The maximum sensitivity and specificity were obtained at a cutoff value of − 251.8 HU.

**Conclusions:**

The evaluation of mCT value has a possibility to predict STAS and may potentially contribute to the selection of suitable treatment strategies.

## Background

With the widespread use of low-dose CT screening for lung cancer, more early lung adenocarcinomas have been detected [1]. For stage IA lung adenocarcinoma, radical resection is still the preferred and recommended treatment according to guidelines [2]. The heterogeneity of malignant tumors still leads to differences in long-term prognosis after surgery [3]. Suzuki et al. [4] has reported that less-invasiveness stage IA lung adenocarcinoma may be more suitable for sublobar resection. Therefore, recognizing less-invasiveness stage IA lung adenocarcinoma prior to surgery has become a significant challenge for thoracic surgeons [5].

Spread through air spaces (STAS) is an extension of primary lung cancer in which tumor cells extend beyond the tumor margin into the alveolar space, and has been reported to occur 15–55% of the time in patients with early-stage lung adenocarcinoma [6, 7]. Furthermore, studies of non-small cell lung cancer have shown that the presence of STAS is an important predictor of recurrence, particularly in patients who have undergone surgical procedure [8]. Therefore, preoperative prediction of the presence or absence of STAS is important in determining the treatment strategy, including the surgical approach for patients with early-stage lung adenocarcinoma.

Quantitative densitometric methodologies, and mean computed tomography (mCT) value have been reportedly used to evaluate ground-glass opacity (GGO) lesions. We previously reported that the mCT value of GGO lesions is a risk factor associated with their future change [9], and the evaluation of mCT value is useful in predicting less invasive lung cancer [10]. There have been no studies regarding the use of mCT values in lung cancer and the presence of STAS, which can be of great significance for treatment decisions.

The objectives of this study were to investigate the usefulness of using mCT value for predicting STAS, and to assess whether they contribute to determining the benefits of performing a limited resection.

## Methods

### Patients and data collection

This study was approved by our hospital’s internal review board. Between March 2020 and November 2022, 167 consecutive patients underwent pulmonary resection for lung cancer. From these patients, those diagnosed with clinically without lymph node metastasis (cN0) and having a pathological diagnosis of adenocarcinoma after undergoing surgery were included in this study. We excluded cases with multiple primary lung cancers, without PET/CT data and STAS data. We reviewed their medical records, including the results of pathologic examination. For each case, the surgical specimens were reviewed and classified according to the latest 2015 WHO classification criteria for lung adenocarcinoma [11]. The primary endpoint of the study was the presence of STAS as diagnosed by postoperative pathology.

### Image acquisition and analysis

#### CT scanning

**Fig. 1:**
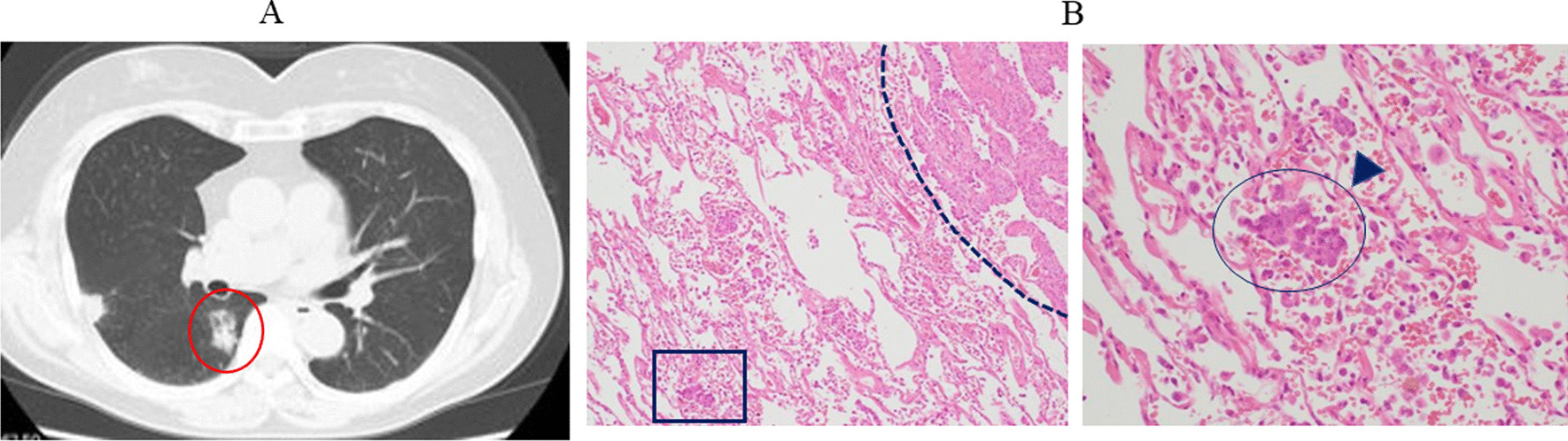
CT and histologic findings of the case with STAS positive. **A** CT shows a partly solid nodule in the right lower lobe. The m-CT value was − 251.8 HU (red circle). **B** Histologic figures of spread through air spaces (STAS) with floating clustures (arrows) of adenocarcinoma cells

CT scans were performed from lung apex to base at mid-inspiration during a held breath using a section thickness of 2.5 mm (Asteion 4, Toshiba, Tokyo, Japan). Contrast agent was not always used, especially for GGO dominant lesions. Two thoracic surgeons with 24 and 11 years of experience independently viewed these images and subjectively classified the nodules. Pure GGO was defined as a shadow that was completely occupied by a hazy area of increased attenuation of the lung, with preserved bronchial and vascular margins of the lesion with no solid regions on high-resolution computed tomography (HRCT). The longest diameters of the GGO lesions and solid portion were measured. The proportion of GGO was calculated using a previously published method [12] and defined as the consolidation/tumor (C/T) ratio. The maximum diameter and one-dimensional mCT values were measured using a computer graphics support system (Synapse® PACS, Fujifilm, Tokyo, Japan). The shape of the region of interest was standardized for each patient and configured by freehand drawing. M.T and M.Y drew the ROI on the graphic support system. The mCT value was evaluated in slice having highest density (Fig. 1A). The multi-observer variation was corrected by calculating the mean value from the two observers.

#### PET/CT scanning

All patients fasted for at least 6 h and were injected intravenously with FDG (3.5 MBq/kg), after which they rested for approximately 60 min. Images from the head to the upper thigh were acquired in the three-dimensional mode at 2 min per bed position with the patient in the supine position using a PET/CT scanner (Discovery MI, GE Healthcare, Waukesha, WI, USA) with a 64-slice CT component. PET/CT was evaluated by the imaging analysis software SAI viewer® (Fuji Medical System, Tokyo, Japan). The maximum standardized uptake value (SUV max) was automatically measured. The SUV max was the maximum value of a volume of interest.

### Pathological examination of STAS

The surgically resected specimens were fixed in 10% formalin, sectioned into slices with a thickness of 4 μm, and stained with hematoxylin and eosin. STAS was defined as tumor cells within alveolar spaces in the lung parenchyma beyond the edge of the main tumor (Fig. 1B). The presence of STAS was evaluated by a pathologist with more than 10 years of diagnostic experience.

### Statistical analysis

Receiver operating characteristics (ROC) analysis was used to compare the ability to predict the presence of STAS using the mCT value, SUVmax value, C/T ratio, solid tumor size. Logistic regression analysis was carried out to investigate potential pretreatment predictors of recurrence. The Fisher exact test was used for the univariate analysis and a logistic regression model for the multivariate analysis. The 95% confidence interval (95% CI) was calculated and all *p-*values were two-sided. Age, tumor size, solid tumor size, C/T ratio, SUVmax value and mCT value, were all included in the univariate analysis. Since this was a retrospective study, the variables for univariate analysis were selected postoperatively. Variables that can be assessed from medical records, radiologic imaging, nuclear medicine examination that are useful for diagnosing malignant potential were included in the univariate analysis. Univariate factors with a *p*-value of < 0.05 were included in the multivariate analysis. All data regarding continuous variables were expressed as mean ± SD. Significant differences were evaluated using the *t*-test for continuous variables and the chi-square -test for categorical variables. Analyses were performed using the JMP Pro (Ver.12) (SAS Institute, Inc, Cary, NC). A p -value of < 0.05 was considered statistically significant.

## Results

**Fig. 2:**
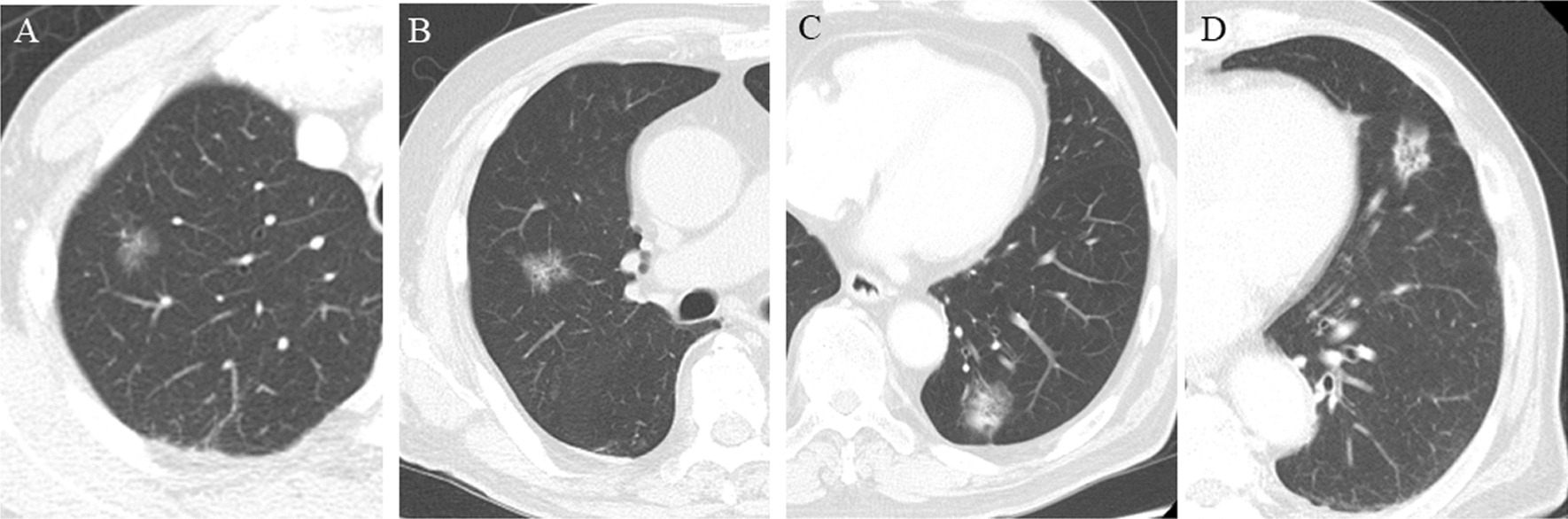
Comparison of the mean computed tomography (m-CT) values. The m-CT value was **A** − 701.8, **B** − 530.4, **C** − 462.5, **D** − 295.1 Housfield units (HU)

**Fig. 3:**
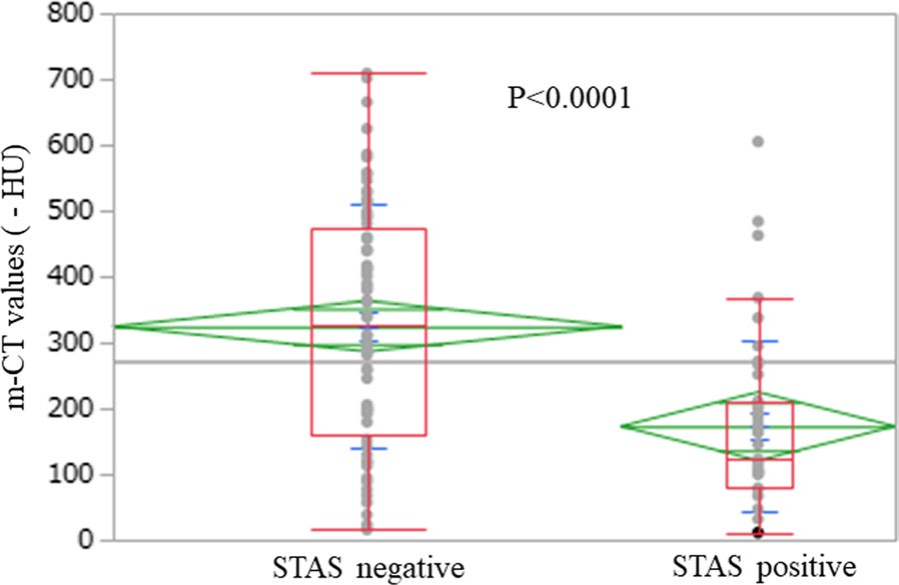
Distribution of the mean computed tomography (m-CT) values in Housfield units (HU) between STAS negative and STAS positive groups

**Fig. 4:**
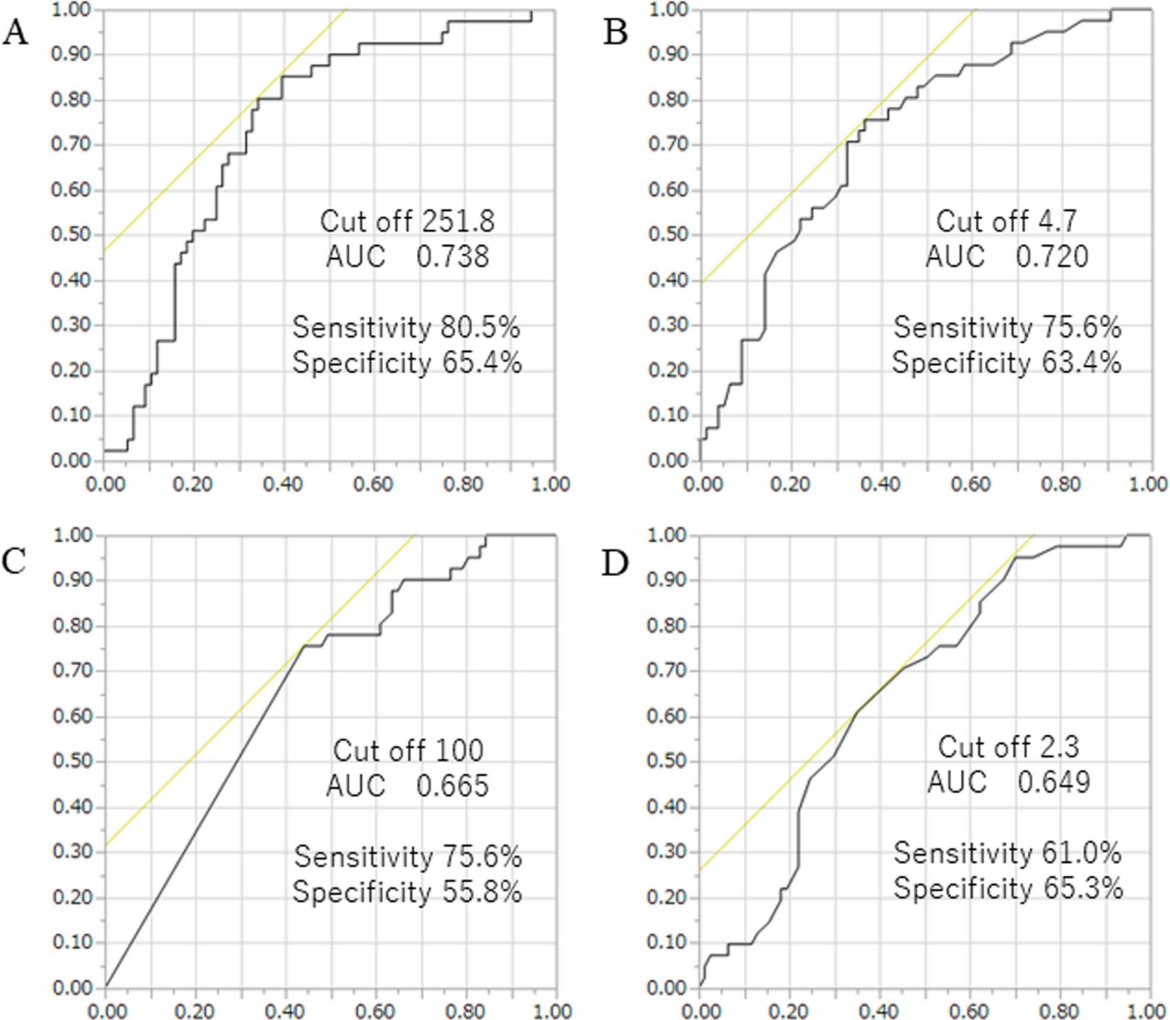
Receiver operating characteristics curves predicting the STAS. **A** mean CT value. Cut-off value: 251.8, AUC: 0.738, Sensitivity: 80.5%, Specificity: 65.4%. **B** SUVmax value. Cut-off value: 4.7, AUC: 0.720, Sensitivity: 75.6%, Specificity: 63.4%. **C** consolidation/tumor ratio. Cut-off value: 100, AUC: 0.665, Sensitivity: 75.6%, Specificity: 55.8%. **D** solid portion size. Cut-off value: 2.3, AUC: 0.649, Sensitivity: 61.0%, Specificity: 65.3%

The CT findings of various nodule lesions are presented in Fig. 2. Figure 2A, B shows a typical pure GGO lesion. In Fig. 2C, D, the tumors are homogeneous in density, but are too dense to be considered pure GGO; therefore it is difficult to calculate the C/T ratio. The mCT values were − 324.9 ± 19.3 HU for STAS negative group and − 173.0 ± 26.3 HU for STAS positive group (*p* < 0.0001) (Fig. 3). Clinico-pathological characteristics are shown in Table 1. A total of 118 patients met both clinical and imaging criteria for inclusion in this study. Of these, 63 were men and 55 were women. Their ages ranged from 46 to 92 years, with a median of 73 years. Clinical T1 stage accounted for 80.5%. Four cases were upstaged to pathological N1 and 11 cases were upstaged to pathological N2. Forty-one lesions (34.7%) out of 118 lesions were positive for STAS. The comparison of clinico-radiological data between lesions in STAS negative and STAS positive are summarized in Table 2. The STAS positive group was strongly associated with a high mCT value, high C/T ratio, large solid tumor size, large tumor size and high SUVmax value. Among 41 STAS positive cases, 31 cases were pure solid and 10 cases were part-solid cases. We attempted to predict the presence of STAS based on the mCT value, SUVmax value, C/T ratio, solid tumor size, and ROC curve analysis was performed to determine the appropriate cutoff value (Fig. 4). The maximum sensitivity and specificity were obtained at a cutoff value of − 251.8 HU, 4.7, 100%, 2.3 cm, respectively. The ROC area under the curve value of the mCT value was the highest (0.738; 95% CI 0.78–0.94), followed by SUVmax value (0.720; 95% CI 0.74–0.91), C/T ratio (0.665; 95% CI 0.79–0.86), solid tumor size (0.649; 95% CI 0.68–0.87). Correlation coefficients between these factors were as follows, mCT value versus SUVmax value: r = − 0.57, p = 0.21; mCT value vs C/T ratio: r = − 0.76, p < 0.001 and mCT value vs solid size: r = − 0.60, p = 0.07. Table 3 shows the results of univariate and multivariate analyses for predicting STAS. Clinical T stage, mCT value and SUVmax value were selected for the multivariate analysis, but ruled out C/T ratio because relatively strong correlation could be found between mCT value (r = − 0.76). Multiple logistic regression analyses using the preoperatively determined variables revealed that mCT value (*p* = 0.015) are independent predictive factor of predicting STAS. However, clinical T stage and SUVmax valuewere not statistically significant (*p* = 0.42, 0.49, respectively).

**Table 1:** Clinico-pathological characteristics

Factors	Number range	%
*Gender*		
Male	63	53.4
Female	55	46.6
*Age*		
Range	46—92	
Mean ± SD	73.7 ± 7.1	
*Whole tumor size (cm)*		
Range	0.8 – 7.8	
Mean ± SD	2.6 ± 1.2	
*Solid tumor size (cm)*		
Range	0.3 – 6.4	
Mean ± SD	2.2 ± 1.3	
*SUV max*		
Range	0.8 – 17.1	
Mean ± SD	6.1 ± 4.4	
*Operative procedure*		
Partial resection	19	16.1
Segmentectomy	26	22
Lobectomy	73	61.9
*Clinical T stage*		
T1mi	6	5.1
T1a	16	13.5
T1b	38	32.2
T1c	35	29.7
T2a	12	10.2
T2b	4	3.4
T3	7	5.9
*Pathological T stage*		
T1mi	13	11
T1a	12	10.2
T1b	38	32.2
T1c	23	19.5
T2a	20	16.9
T2b	3	2.5
T3	9	7.6
*Pathological N stage*		
N0	103	87.3
N1	4	3.4
N2	11	9.3

SD: standard deviation

**Table 2:** Comparison of clinico-radiological data between lesions in STAS(-) and STAS ( +) categories

Factors	STAS (−) (n)	STAS (+) (n)	p value
*Gender*			0.83
Male	41	21	
Female	36	20	
*Age*			0.06
< 68	11	12	
≥ 68	66	29	
*Tumor size*			0.08
< 2.3 cm	33	11	
≥ 2.3 cm	44	30	
*Solid size*			0.007
< 2.3 cm	50	16	
≥ 2.3 cm	27	25	
*C/T ratio*			0.009
< 100	43	10	
100	34	31	
*mCT value (H.U)*			< 0.001
< -251.8	51	8	
≥ -251.8	26	33	
*SUV*			< 0.001
< 4.7	49	10	
≥ 4.7	28	31	

*n* number of cases, *STAS*(−) without STAS, *STAS*(+) with STAS, *HU* Hounsfield unit, *C/T ratio* consolidation/tumor ratio, *mCT* mean computed tomography, *SUV* standardized uptake value

**Table 3:** Multiple logistic regression analysis predicting STAS

Risk factor	Univariate analysis				Multivariate analysis			
	Odd ratio	95% CI		P-value	Odd ratio	95% CI		P-value
Age	2.2	0.18	2.76	0.53				
Tumor size	0.46	0.05	4.1	0.47				
Solid size	0.75	0.56	1.01	0.056				
Clinical T stage	2.87	1.32	6.41	0.007	0.38	0.99	2.12	0.42
C/T ratio	0.96	0.94	0.99	0.006				
mCT value (H.U)	5.32	8.7	45.4	< 0.0001	1.99	1.18	12.8	0.015
SUVmax value	3.84	1.03	2.54	0.001	0.31	0.92	1.19	0.49

*CI* confidence interval, *C/T ratio* consolidation/tumor ratio, *mCT value* mean computed tomography value, *SUV* standardized uptake value, *H.U* housfield unit

## Discussion

The present study aimed to evaluate the relationship between STAS and the clinic- radiological factors of patients with clinical N0 lung adenocarcinoma. Evaluation of various CT features, such as the mCT values, C/T ratio, tumor size and SUV value of FDG-PET can be helpful in predicting the presence of STAS. In particular, we initially demonstrated that the mCT value of the GGO lesion is a sensitive marker for predicting STAS in clinical N0 lung adenocarcinoma.

The phenomenon of spread through air spaces (STAS) which was first identified by Kadota et al. [13], is defined as the spread of lung cancer tumor cells into the air spaces of the lung parenchyma adjacent to the main tumor. Recent studies on NSCLC have shown that STAS is a significant risk factor for recurrence and a prognostic factor for poor OS, especially after sublobar resection [8, 14–16]. STAS not only affects patient prognosis but also influences the choice of surgical approach. Therefore, assessing the risk of STAS in the primary tumor through preoperative radiological imaging can provide important auxiliary diagnostic information for thoracic surgeons in selecting surgical approaches and can impact patient prognosis.

If we could predict the presence of STAS in the primary tumor through preoperative CT imaging, it would be very helpful for thoracic surgeon in selecting surgical approach. In a previous study, STAS was significantly related to solid nodules on computed tomography [17].

Currently, there are several clinical studies about C/T ratio with STAS and confirmed that the C/T ratio was associated with STAS positive tumors [18, 19]. In a previous study, STAS was significantly related to 18F FDG-PET findings, especially SUVmax value [20] and MTV/CTV ratio [21]. Kim et al. reported that STAS was more common in solid tumors than in part-solid or ground-glass lesions [18]. In the presented study, 10 out of 53 part-solid lesions and 31 out of 65 solid lesions revealed STAS. In subgroup analysis among part-solid nodules, SUVmax was significantly higher in STAS positive group comparing STAS negative group. On the other hand, there was no significant difference between the presence of STAS and PET parameters among solid nodule lesions.

With recent advances in diagnostic imaging technologies, GGO lesions are increasingly detected using HRCT scans [22]. In a clinical setting, several types of GGO can be encountered. Suzuki et al. [23] classified peripheral small-sized adenocarcinoma into six categories and reported that the classification was significantly associated with pathologic prognostic factors. It is difficult to measure the size of the solid part of the tumor when the nodule comprises a heterogeneous mixture of GGO and solid tumor. The mCT value is useful for tumors that are too dense to be called pure GGO but are not pure solid tumor. The mCT value adds a diagnostic value to the C/T ratio for tumors for which the size of solid portion is difficult to measure. Quantitative densitometric methodologies, and mCT value have been reportedly used to evaluate GGO lesions [24, 25]. We previously reported that the mCT value of GGO lesions is a risk factor associated with their future change [9], and the prediction of recurrence [26].

This study has several limitations. Firstly, it is a single-center retrospective study, which may have some selection bias, and lacks prospective validation. Secondly, the sample size needs to be further expanded.

## Conclusions

The evaluation of the mCT value has a potential to predict STAS preoperatively. For such as patients with adenocarcinoma manifested as mixed GGO, added new information to the C/T ratio, and may contribute to establish the optimal treatment strategies for this disease.

## Data Availability

The dataset supporting the conclusions of this article is available upon request.

## Abbreviations

SUVmax value: Maximum standardized uptake value
18F FDG-PET: F-18 fluorodeoxyglucose positron emission tomography
STAS: Spread through air spaces
mCT value: Mean computed tomography value
GGO: Ground-glass opacity
HRCT: High-resolution computed tomography
ROC: Receiver operating characteristics
C/T ratio: Consolidation/tumor ratio
HU: Housfield units
